# Research progress in the radioprotective effect of the canonical Wnt pathway

**DOI:** 10.7497/j.issn.2095-3941.2013.02.001

**Published:** 2013-06

**Authors:** Jin-Feng Wang, Chao Liu, Qu Zhang, Guan-Hong Huang

**Affiliations:** 1 Department of Radiation Oncology, Bengbu Medical College, Bengbu 233000, China;; 2 Department of Radiation Oncology, The Second People’s Hospital of Lianyungang, Lianyungang 222000, China;; 3 Department of Emergency Surgery, Bengbu Medical College, Bengbu 233000, China;; 4 Department of Emergency Surgery, Anhui Oncology Hospital of Bengbu Medical College, Bengbu 233000, China

**Keywords:** Signaling transduction, canonical Wnt pathway, β-catenin, radioprotection, radiation

## Abstract

Irradiation from diverse sources is ubiquitous and closely associated with human activities. Radiation therapy (RT), an important component of multiple radiation origins, is a common therapeutic modality for cancer. More importantly, RT provides significant contribution to oncotherapy by killing tumor cells. However, during the course of therapy, irradiation of normal tissues can result in a wide range of side effects, including self-limited acute toxicities, mild chronic symptoms, or severe organ dysfunction. Although numerous promising radioprotective agents have emerged, only a few have successfully entered the market because of various limitations. At present, the widely accepted hypothesis for protection against radiation-caused injury involves the Wnt canonical pathway. Activating the Wnt/β-catenin signaling pathway may protect the salivary gland, oral mucosa, and gastrointestinal epithelium from radiation damage. The underlying mechanisms include inhibiting apoptosis and preserving normal tissue functions. However, aberrant Wnt signaling underlies a wide range of pathologies in humans, and its various components contribute to cancer. Moreover, studies have suggested that Wnt/β-catenin signaling may lead to radioresistance of cancer stem cell. These facts markedly complicate any definition of the exact function of the Wnt pathway.

## Introduction

**Figure 1:**
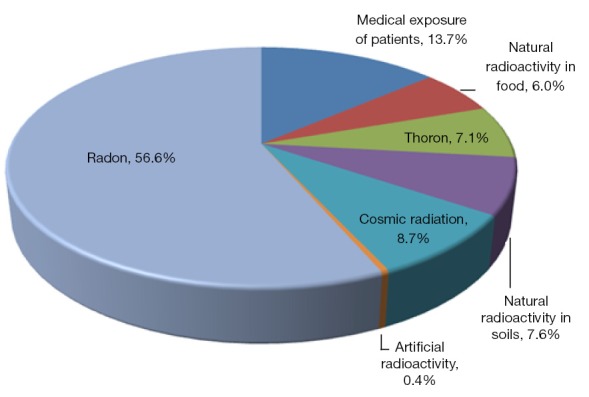
Dose contribution to the individual radiation absorption from all sources of radiation. Artificial radiation consists primarily of medical exposure of patients, accounting for approximately 14% of the individual radiation absorption and has been attracting more and more attention over recent years.

Irradiation from diverse sources is ubiquitous and is closely associated with human activities. Among all sources of radiation (**Figure 1**^1^), natural radiation, including radon, thoron, cosmic radiation, and natural radioactivity in soil and food, dominates the average dose of individual radiation adsorption. The dose from natural radiation can not be controlled. However, artificial radiation, which consists primarily of medical exposure of patients, accounts for approximately 14% of individual radiation absorption and has been attracting increasing attention in recent years. As one of the treatments for malignant tumors, radiation therapy (RT) is administered to more than half of all cancer patients during the course of their treatment^2^, according to the 2001 statistics from the Swedish Council on Technology Assessment in Health Care. Local or regional control of malignancies can be achieved either through RT alone or in combination with other modalities, such as chemotherapy and surgery. However, during the course of therapy, the irradiation of normal tissues can result in a wide range of side effects, including self-limited acute toxicities, mild chronic symptoms, and severe organ dysfunction.

Hence, prevention or treatment of early and late RT effects will improve quality of life and may even increase cancer curability by intensifying the regimens of therapy. Moreover, a large pool of chemical and molecular agents can be effective in protecting and treating radiation damage^3^. In recent years, technological improvements in radiation delivery and chemical modifiers for radiation injuries have been used to reduce toxicities associated with therapeutic radiation. Although improvements have been achieved in this regard, radiation damage remains a limiting factor in treating numerous diseases with RT. Thus, the search for techniques that can protect normal tissues from radiation has been an area of intense investigation.

Despite the fact that numerous promising radioprotective approaches are emerging, to date, amifostine (WR2721) remains to be the only drug recommended as an effective radioprotective agent against xerostomia resulting from irradiation^4^^,^^5^. However, the use of this drug is limited because of its numerous side effects including nausea, cutaneous reactions, and hypotension, etc. Therefore, the search for radioprotective agents with high potency and low toxicity should be a primary concern for further research.

The Wnt signal transduction pathway (shorten as the Wnt pathway hereafter) is an evolutionarily conserved signaling pathway with an important role in fetal development and central nervous system formation, as well as in cell growth, migration, and differentiation^6^^-^^8^. Recently, an *in vivo* research^9^ reported that concurrent transient activation of the Wnt/β-catenin pathway could prevent radiation-induced salivary gland dysfunction. In the study of Thotala *et al.*^10^, Wnt signaling was found to play a central role in renewing damaged intestinal epithelium. Furthermore, another research^11^ demonstrated that upregulation of the Wnt/β-catenin pathway could accelerate repair of radiation oral mucositis.

These pieces of evidence indicate that the Wnt/β-catenin pathway has a significant role in radioprotection. However, the deregulation of the Wnt/β-catenin pathway has been proposed as one of the signaling pathways responsible for carcinogenesis in the hematopoietic systems, the intestines, and the epidermis, as well as for chronic myelogenous leukemia, lung cancer, colorectal cancer and gastrointestinal cancer (GI)^12^. Studies have also identified overexpression of the Wnt signaling pathway in malignant breast cell lines^13^. Moreover, Wnt signaling is involved in cancer stem cell radioresistance^14^. These facts markedly complicate any definition for the exact function of the Wnt pathway. Thus, further investigation regarding the detail of this pathway is highly important to contribute to the development of radioprotection. In this review, our first objective is to describe radioprotective studies regarding the effect of the Wnt/β-catenin pathway to radiation-induced salivary gland dysfunction, oral mucositis, and GI syndrome. Our second objective is to emphasize the potential roles of the Wnt/β-catenin pathway in cancer development. We expect that Wnt signaling in different contexts will facilitate the improvement of RT.

## Radiation damage

Tissues are well organized and composed of cells with epithelial and connective tissue origins. Tissue regeneration ability varies with the cell source. First, continuously regenerating cells consist of the epithelial and hematopoietic bone marrow cells. Researchers have shown that the doubling time for regenerating clonogenic cells in the crypts of the small intestine is less than one day, and that the entire intestinal tract epithelial compartment renews completely within a few days^15^. Second, fibroblasts and the endothelial vascular cells are slow-renewing cells. Studies have reported that the endothelial cell *in vivo* turnover in conduit blood vessels is approximately one to several years, with only 0.1% of cells actively proliferating in quiescent vessels^16^. Lastly, common knowledge suggests that neurons never proliferate.

**Figure 2:**
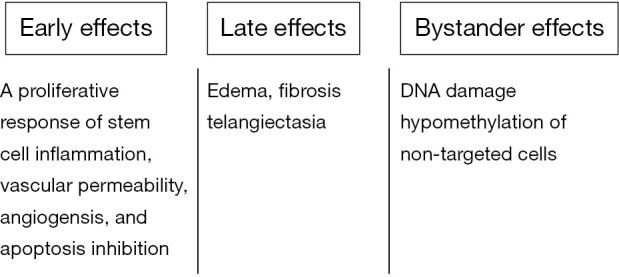
Type of radiation damage.

Given such distinct cellular compartmentalization, understanding the events occurring during and shortly after irradiation of tissues and cell is essential. This information is necessary in understanding the mechanisms of radioprotectors and mitigators. **Figure 2** shows the sequence of events in cells and tissues following radiation exposure. Tissue exposure to ionizing radiation results in early radiation effects, late radiation effects, and bystander effects. Early radiation effects include cellular depletion^17^, which involves cellular death and depletion of a tissue followed by a proliferative response of stem cells and reactive gene activation^18^^,^^19^. The latter entails cellular and tissue dysfunction followed by increased vascular permeability, tissue edema, growth factor and cytokine production on behalf of fibroblasts and endothelial cells, and chemoattraction of macrophages and other white cells which leads to radiation inflammation. Chronic tissue dysfunction and disorganization result in late toxicity. Bystander effects^20^, refer to the radiation damage induced in cells within an organ or the entire body that have not been directly exposed to radiation, have recently become the focus of intensive research to clarify their molecular mechanisms. Various radioprotective policies can disturb destructive processes resulting from cell exposure to radiation, including the occurrence of genetic mutation, cell death, and tissue disorganization.

Death pathway modulators, which regulate early radiation damage, are among such radioprotective policies. This policy involves the activation of the Wnt/β-catenin pathways.

## The Wnt signaling pathway

The Wnt signaling pathway was discovered during an embryogenesis experiment on Xenopus laevis. The Wnt1 gene, the first gene of the Wnt protein family, was identified in 1982 as a gene activated by the integration of mouse mammary tumor virus proviral DNA into virally induced breast tumors^21^. Since then, Wnt signaling has often been reported associated with cancer and cell proliferation.

Wnt ligands are a family of secreted glycoproteins involved in cell-to-cell communication that controls each major develop mental process, including cell-fate determination as well as cell proliferation, polarity, adhesion, motility, and apoptosis, and subsequently, patterning and morphogenesis^22^. Historically, Wnt ligands activate two major intracellular pathways: the canonical (β-catenin-dependent)^23^ and non-canonical (β-catenin-independent) pathways^24^. Β-catenin is the main effector of canonical signaling. In this paper, we will thoroughly review the research progress in the radioprotection of the Wnt canonical pathway.

**Figure 3:**
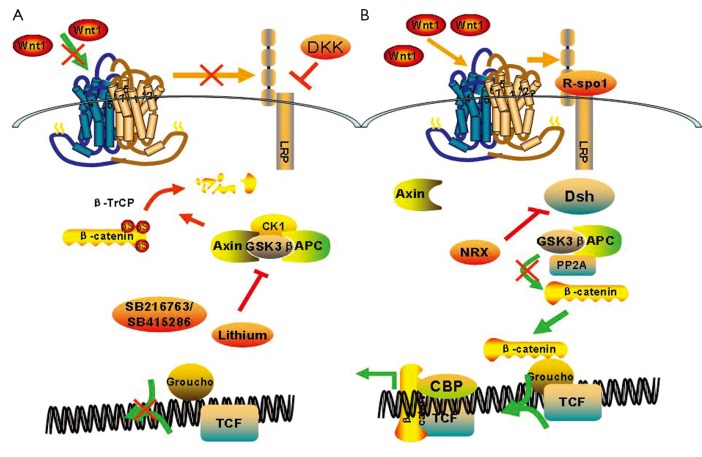
Wnt canonical pathway. (A) In the absence of a Wnt ligand, the cytoplasmic β-catenin is degraded by the “destruction complex”, which contains adeomatous polyposis coli (APC) and axin, glycogen synthase 3β (GSK-3β) and casein kinase 1α (CK1α) phosphorylated β-catenin. Accordingly, phosphorylated β-catenin is recognized by β-TrCP and constantly degraded by the ubiquitin-proteasome pathway; (B) The binding of WNTs, such as WNT3A and WNT1, to frizzled (FZD) and LRP5 or LRP6 co-receptors transduces a signal across the plasma membrane that results in the activation of the Dishevelled (DVL) protein. Activation of Dvl induces the dissociation of GSK-3β from Axin and leads to the inhibition of GSK-3β. This results in the accumulation of β-catenin in the cytoplasm and ultimately the nucleus where it displaces cyclic AMP response element-binding protein (CBP) β-catenin and facilitates transcriptional actiation of lymphocyte enhancer binding actor (LEF)/T cell factor (TCF) resulting in altered gene transcription. Ultimately, Wnt canonical pathway modulates changes in cell behaviours such as proliferation, survival and differentiation. DKK: dickkopf homologue; NRX: nucleoredoxin; R-spon1: R-spondin1.

Cytoplasmic β-catenin is degraded by the “destruction complex” in the absence of a Wnt ligand binding to its receptor complex. In the destruction complex, Axin acts as a scaffold protein, in which adenomatous polyposis coli (APC), glycogen synthase kinase 3β (GSK-3β), and casein kinase 1α (CK1α) bind to facilitate the sequential phophorylation of β-catenin in 45 serine by kinase CK1α and 41’threomine, and in 37’,33’serine by GSK-3β^25^^,^^26^. Accordingly, phosphorylated β-catenin is recognized by β-transducin-repeat-containing protein (β-TrCP) and is constantly degraded by the ubiquitin-proteasome pathway. Wnt signaling is activated via Wnts ligation of the seven transmembrane frizzled (Fz) proteins and the low-density lipoprotein receptors. Cytoplasmic protein disheveled (Dvl) is then recruited, phosphorylated, and activated. Activation of Dvl induces the dissociation of GSK-3β from Axin and leads to GSK-3β inhibition. Phosphorylation and degradation of β-catenin is inhibited as a result of the inactivation of the destruction complex. Subsequently, the stabilized β-catenin translocates into the nucleus. Nuclear β-catenin is the ultimate effector, binding to transcription factor T cell factor and lymphoid-enhancing factor(Tcf/Lef), which leads to changes in different target gene expressions^27^ (**Figure 3**).

With the increasing interest of researchers in the Wnt/β-catenin pathway, Wnt signaling is revealed to be modulated by numerous evolutionarily conserved inhibitors and activators. The Wnt/β-catenin pathway can be specifically blocked by endogenously secreted inhibitors from the Dickkopf (DKK) family, which interact with LRP5/6 and high-affinity receptors from the Kremen family, thus causing rapid endocytosis of Kremen-Dkk-LRP complex and removal of LRP from the cell membranes^28^^,^^29^. The Wnt activator roof plate-specific spondin (R-spondin)^30^^,^^31^ promotes Wnt signaling by binding to Wnt receptors or reloading a Wnt-inhibitory step. Previously, lithium was found to be an activator of the Wnt signaling pathway by inhibiting GSK-3β from species as diverse as *Dictyostelium discoideum and Xenopus laevis*^32^. Moreover, research showed that Wnt/β-catenin signaling is of major importance in controlling lithium-dependent thyrocyte proliferation^33^ and lithium-enhanced bone formation and improves bone mass in mice by activating Wnt/β-catenin signaling. In this study, we focus on the protective effects of the Wnt/β-catenin pathway on radiation-induced salivary gland dysfunction, oral mucositis, and GI syndrome.

### Radioprotection for radiation-induced salivary gland dysfunction

Head and neck cancer (HNC) is the fifth most common cancer, with an estimated annual incidence exceeding 500,000 worldwide^34^. Given that radiation therapy is the most common form of treatment for HNC, non-cancerous salivary gland cells (SGs) are often exposed to radiation. Because of the enhanced radiosensitivity of SGs, irreversible hyposalivation is common (60% to 90%) among HNC survivors treated with radiotherapy^35^. Hyposalivation leads to dental caries and periodontal disease. Moreover, it causes mastication and swallowing problems, a burning sensation in the mouth, and dysgeusia, etc, thus significantly impairing the quality of life of patients^36^. At present, a dry mouth can be temporarily relieved through artificial saliva and saliva secretion stimulators^37^. Irreversible hyposalivation was previously hypothesized to be caused by the sterilization of the primitive glandular stem/progenitor cells that normally continuously replenish aged saliva producing cells^38^. By contrast, recent findings suggest that human salivary gland stem/progenitor cells remain dormant even after irradiation^39^, thus suggesting that the loss of the regenerating capacity after irradiation may be reversible.

To our knowledge, Hakim *et al.*^40^ conducted the first *in vivo* study that investigated the increase of the Wnt-1 expression in irradiated but viable acinar structures along with the membranous upregulation of β-catenin. In accordance with previous *in vitro* studies, Wnt pathway activation was demonstrated to provide a key radioprotective mechanism in irradiated cancer cells^40^^-^^42^.

To further understand the involvement of Wnt signaling in normal SG development and regeneration after injury. Hai *et al.*^43^ used Wnt reporter transgenic mice and discovered that Wnt signaling is active in the ductal regions of postnatal submandibular glands (SMGs) and is upregulated with Hedgehog (Hh) signaling during SMG regeneration induced by the ligation of the main secretor ducts. Inhibiting of epithelial Wnt signaling increases SMG stem/progenitor cell population and activates Hh signaling. The results of this previous study indicated that Wnt/beta-catenin signaling regulates the activity of salivary gland stem/progenitor cells upstream of the Hh pathway, and suggested that manipulating Wnt or Hh signaling is a potential strategy for restoring salivary function in patients with hyposalivation.

To further explore the potential of activating Wnt/β-catenin signaling in preventing radiation damage to salivary glands, Hai *et al.*^9^ used Wnt reporter transgenic mice exposed to 15 Gy single-dose radiation in the head and neck area and obtained the following results: (1) radiation damage did not significantly affect the activity of the Wnt/β-catenin pathway compared with physical damage; (2) transient expression of the Wnt1 pathway prevented chronic salivary gland dysfunction following irradiation by suppressing apoptosis and preserving functional salivary stem/progenitor cells; (3) excessive Wnt activation before irradiation failed to inhibit apoptosis probably because of the extensive induction of mitosis and the upregulation of the proapoptosis gene PUMA, whereas activation after irradiation probably missed the critical treatment window.

### Radioprotection for radiation-induced oral mucositis

Oral mucositis, which is characterized by mucosal damage and inflammation in the oral cavity, is a common complication of chemotherapy and radiotherapy among cancer patients with head and neck tumors and hematological malignancies^44^. The impairment of the regenerative capability of the oral mucosal epithelium leads to atrophy and ulceration, and further limits the ability of patients to tolerate optimal anti-tumor treatment regimens^43^. As such finding an effective prevention method for radiotherapy-induced oral mucositis is urgently needed to enhance treatment effect and improve the quality of life of patients with head and neck tumors. The keratinocyte growth factor (KGF) which is one of the mucosal epithelial growth factors has been shown to be efficient in treating experimental animal models with chemotherapy or radiotherapy-induced oral mucositis. This growth factor is also the first approved biologic therapy for human cancer patients with oral mucositis. However, KGF has been reported to have numerous side effects^45^^,^^46^.

The R-spondin family of proteins comprises novel secreted proteins acting as major agonists and modulators of the Wnt-β-catenin signaling pathway^47^^,^^48^. Four human paralogs^49^, each containing a lead signal peptide; two cystein-rich, furin-like domains; and one thrombospondin type 1 domain human R-spondin-1 (Rspo1), a 29 kd, 263 amino acid protein, have been shown to augment canonical Wnt/β-catenin signaling in both *in vitro* and *in vivo* studies^50^. Recently, R-spondin has been found to exhibit synergistic cooperation with Wnt ligands in activating canonical Wnt signaling. The Wnt/β-catenin signaling has a pivotal role in both embryonic development and homeostatic self-renewal of adult tissues, including GI and oral mucosa. Zhao *et al.*^11^, using transgenic Wnt reporter TOPGAL mice, identified the oral mucosa as a target tissue for Rspo1 by injecting recombinant Rspo1. Their results demonstrated that Rspo1 significantly reduces both chemotherapy- and radiotherapy-induced damage to the oral mucosa by amplifying Wnt/β-catenin signaling and subsequently triggering epithelial cell growth to accelerate mucosal healing in mice. Moreover, Rspo1 decreases the extent of overt tongue ulceration by repairing disintegrated mucosa in mice exposed to high doses of radiation delivered to the head region. Rspo1 is effective in both injury and healing phases of oral mucositis in mice by increasing basal layer epithelial cell density in the tongue. More importantly, using Rspo1 appears to be effective during various stages of experimental oral mucositis in mice.

The LRP5/6 receptor also appears to play a role in activating the Wnt/β-catenin pathway. Wei *et al.*^51^ investigated the combination of Rspo1 with LRP6 and Fzd8. Their results showed that Rspo1 combined firmly with LRP6, but hardly combined with Fzd8. Therefore, LRP6 was considered to be an affinity receptor of Rspo1 protein.

In summary, Rspo1 demonstrates a protective effect against radioactive oral mucosa inflammation through the activation of the Wnt/β-catenin pathway. As a receptor protein for the Wnt signaling pathways, the internalization of LRP6 is an essential step in β-catenin activation^52^. However, factors affecting Rspo1 and LRP6 combination as well as the improvement of their binding ratio still need to be elucidated.

### Radioprotection for radiation-induced GI syndrome (RIGS)

Normal homeostasis of the intestinal epithelium is a dynamic balance maintained by an intricate cell replacement process in which terminally differentiated epithelial cells are continuously and rapidly replaced by replication and differentiation of epithelial cells (transit cells) located within the intestinal crypt. RIGS results from a combination of direct cytocidal effect on the intestinal crypt and on endothelial cells, and the subsequent loss of the mucosal barrier. This situation results in electrolyte imbalance, diarrhea, weight loss, infection, and mortality. DNA damage and, presumably, endoplasmic reticulum and mitochondrion damage induced by radiation-free radicals immediately trigger the pro-apoptotic cell response^53^. GI symptoms, which affect quality of life, can induce change in one or more specific physiological functions in extensively separated parts of the GI tract. Furthermore, these symptoms, which are experienced after pelvic radiotherapy, are substantially widespread and frequently poorly managed^54^. The intestine exhibits strong sensitivity to radiation, which is, in part, caused by continuously renewing cells within the crypt of Lieberkuhn^54^. Radiation-induced apoptosis occurs predominantly within the stem cell region. To date, few studies have elaborated on the molecular determinants of intestinal radiosensitivity. In the GI system, ionizing radiation causes a dose-dependent increase in apoptosis in the small intestinal crypts within hours after exposure^51^. The Wnt/β-catenin pathway has been implicated in regulating radiation-induced apoptosis in crypt cells.

The β-catenin/TCF signal transduction pathway most probably has a significant role in regulating the proliferation and differentiation of intestinal epithelial cells during the regeneratiation and maturation processes along the crypt-villus axis^55^. Wnt signaling and β-catenin activation are important in the proliferation of the pluripotent stem cell which give rise to crypt epithelial progenitors. The amount of Wnt proteins in intestinal epithelial cells decreases with the progression toward the villus. As Wnt signaling decreases, β-catenin forms a complex with APC and Axin (destruction complex), thus leading to the degradation of β-catenin^56^. Therefore, Wnt signaling has a critical role in maintaining the undifferentiated state of intestinal crypt progenitor cells^57^. Barker *et al.*^58^ reported that a Wnt target gene, Lgr5 (that is, leucine-rich-repeat-containing G-protein-coupled receptor 5, also known as Gpr49) was identified as a marker for intestinal stem cells because it marks small columnar cells at the base of the crypt interspersed among Paneth cells. Moreover, research showed that Rspo1 was radioprotective against RIGS and that the mechanism behind this action was probably related to induction of the Wnt/β-catenin pathway.

GSK-3 located upstream of Wnt signaling, is involved in numerous important biological signal paths, and has an essential role in the Wnt/β-catenin pathway. In addition, GSK-3β, a type of GSK-3, is a major component of Wnt signaling which has an important role in the development and renewal of the intestinal epithelium by maintaining stem/progenitor cells and controlling migration and localization of epithelial cells along the crypt-villus axis^59^. Moreover, GSK-3β signaling is a key regulator of radiation-induced injury, and reports have shown that the small molecule inhibitor of GSK-3β has a protective effect against irradiated hippocampal neurons from apoptosis, and consequently, improves cognitive performances in irradiated mice^60^.

Thotala *et al.*^10^ used C57/BL6 mice to evaluate the radioprotective effect of GSK-3β inhibitors. Their results showed that mice pretreated with SB216763 or SB415286 exhibited significant reduction in the number of TUNEL- and Bax-positive cells and an increase in the number of Bcl-2-positive cells in intestinal crypts at 4 h and 12 h after radiation with 4 Gy and 8 Gy, respectively, compared with exposure to radiation alone, without significantly improving the survival rate of mice irradiated with 8 and 12 Gy. Several pathways have been implicated in the targeted molecular radioprotector of GSK-3β inhibitors, including the Bax/caspasae-3 pathway. Alternatively or additively, Wnt signaling transduces the radioprotective effect of GSK-3β inhibitors to perform one of the central roles in renewing damaged intestinal epithelium^59^. Given that GSK-3β phosphorylates β-catenin and targets it for ubiquitination and subsequent degradation, inhibiting GSK-3β can prevent β-catenin phosphorylation and degradation, whereas β-catenin accumulation and activation can promote cell cycle entry and the progression of stem cells within the intestinal crypts.

### The role of Wnt/β-catenin signaling in cancer cells

The Wnt signaling pathway controls myriad biological phenomena throughout the development and adult life of all animals. In parallel, aberrant Wnt signaling underlies an extensive range of pathologies in humans, with its various components contributing to cancer development. Loss of APC function resulting from sporadic colorectal cancer leads to inappropriate stabilization of β-catenin and the formation of constitutive complexes between β-catenin and the intestinal TCF family member TCF4^61^. In addition to APC mutations, factor 7-like 2 (TCF7L2, previously known as TCF4), β-catenin and Wilms tumour gene on the X chromosome have also been predicted to activate Wnt/β-catenin signalling^62^, as shown by the sequencing of colorectal tumors. In addition, mutations that are predicted to disrupt phosphorylation and degradation of β-catenin are frequent in hepatocellular carcinoma (HCC)^63^^,^^64^, medulloblastoma^65^, and ovarian cancer^66^; whereas deletions and truncation mutations in Axin1 are common in HCC and colorectal tumors^67^^,^^68^ (**Table 1**).

**Table 1:** Somatic mutation in WNT pathway genes in various cancers types*

Gene	Type of mutation	Primary tissues	Number of mutated samples	% mutated	Total samples
*APC*	Primarily frameshift and deletion mutations leading to compromised ability to degrade ATNNB1	Large intestine	2,152	39%	5,517
		Stomach	129	15%	214
		Soft tissue	50	12%	430
		Small intestine	34	16%	214
		Pancreas	26	14%	184
		Liver	11	12%	94
*CTNNB1*	Mutations in CTNNB1 cluster around the amino-terminus and prevent the phosphorylation amino acids, S33, S37, T41 and S45, resulting in impaired degradation of CTNNB1	Liver	907	23%	3,933
		Soft tissue	673	42%	1,601
		Endometrium	218	20%	1,098
		Kidney	168	14%	1,225
		Pancreas	125	26%	476
		Ovary	104	11%	913
		Adrenal gland	100	19%	534
		Pituitary	86	24%	360
		Biliary tract	43	10%	433
*AXIN1*	Many mutations prevent AXIN1 from acting as a scaffold to degrade CTNNB1	Biliary tract	10	38%	26
		Liver	49	11%	448
WTX (also known as FAM123B)	Predicted to be loss-of-function mutations	Kidney	125	13%	949
		Large intestine	19	13%	151
*TCF7L2*	Unknown	Large intestine	13	28%	47

*Cited from the Catalogue of Somatic Mutations in Cancer (COSMIC) database.

Genes mutated in at least 10% of the analysed samples for each cancer type are included in the table: APC, CTNNB1β-catenin, and WTX.

Recently, several studies have investigated the possibility of using the Wnt/β-catenin signaling pathway for overcoming radiation resistance. Che *et al.*^69^ described the implication of the Wnt pathway in esophageal cancer. These researchers used a COX-2 inhibitor, that is, NS398, to evaluate its radiosensitizing effect on radioresistant esophageal cancer Eca109R50 Gy cells. NS398 can enhance radiosensitivity of Eca109R50 Gy cells obtained through fractional irradiation from Eca109 cells using several mechanisms, including redistribution of cell cycle, inhibition of DNA-dependent protein kinase catalytic sub-unit expression, and induction of tumor cell apoptosis. The same team reported that NS398 intake could enhance the radiosensitivity of Eca109R50 Gy cells, while decreasing β-catenin expression. In addition, increasing doses was accompanied by an increase in β-catenin^70^ at the molecular level. Kendeziorra *et al.*^71^ reported over-expression of the Wnt transcription factor TCF4 in rectal cancer cell lines that were resistant to chemoradiotherapy.

### Wnt/β-catenin signaling and reactive oxygen species

Radiation is widely believed to affect the homeostasis of reactive oxygen species (ROS)^72^, and that ROS accumulation can cause injury to cells. Numerous studies have indicated that ROS, which are cellular by-products of respiration, can modulate various signaling pathways and have certain physiological roles. Ionizing radiation can induce abnormal levels of ROS, thus exceeding the ability of organisms to clear them and consequently leading to oxidative stress-mediated damaging effects^73^. Recent studies have revealed that superoxide dismutase is expected to play an indispensable role in treating ROS-mediated normal tissue injuries originating from radiation exposure. Although ROS are widely recognized for their role as intracellular messengers, the mechanism of ROS-dependent signaling remains unknown. Previous research has demonstrated that ROS can modulate signaling by the Wnt/β-catenin pathway^74^. The canonical Wnt/β-catenin signaling, which is receiving the most attention, is characterized by the translocation of β-catenin to the nucleus as well as the subsequent downregulation and upregulation of epithelial and mesenchymal markers, respectively. Arnold *et al.*^75^ showed that ROS increased to a certain extent can stimulate cell proliferation and transformation to a certain extent. Moreover, the study of Funato *et al.*^76^ suggested that such enhanced proliferation is at least mediated in part by the release of the nucleoredoxin-dependent inhibition of Wnt/β-catenin signaling. Thus, β-catenin may be a key regulator in determining whether cells will proliferate or arrest to repair oxidative damage.

To our knowledge, the Wnt/β-catenin pathway is an important signaling mechanism for cell proliferation. β-Catenin migrates to the nucleus after the inactivation of GSK-3β, which is followed by the gene expression of cyclin D1^77^. ROS have been known to modulate signaling by the Wnt/β-catenin pathway. Current data show that tempol-elicited downregulation of nuclear β-catenin may indicate that ROS have activated the Wnt signaling/β-catenin pathway in the neural stem/progenitor cells^78^. Further study needs to be conducted to verify the scavenger role of ROS in Wnt signaling pathway.

## Conclusion

The Wnt/β-catenin pathway evidently has an important role in preventing damage induced by RT. The activation of the Wnt/β-catenin pathway, which provides a key radioprotective mechanism in irradiated cancer cells, exhibits protective effect against RT-induced oral mucositis and xerostomia. Moreover, activating Wnt/β-catenin signaling is a potential strategy for treating GI radiation syndrome. As a common signal pathway in the development and adult life of all animals, the Wnt signal transduction cascade, particularly the canonical Wnt pathway, is expected to occupy an indispensable position in treating normal tissue injuries, resulting from radiation exposure. Wnt/β-catenin signaling is associated with the development, renewal, and regeneration of numerous endoderm-derived organs; and research on this field has been ongoing for almost 40 years. Although a number of positive outcomes regarding the activation of the Wnt/β-catenin signaling pathway of antiradiation have been obtained, studies on the activation of the Wnt/β-catenin pathway related to antiradiation research remain extremely few to date. No drug based on Wnt/β-catenin signaling has been approved for radioprotective use in the clinical setting. The canonical Wnt pathway has been linked to cell proliferation in a variety of tissues and systems. Rspo1 is the only protein that can increase the mucosal thickness and reduce ulceration in the oral mucosa after irradiation and chemotherapy, presumably by increasing the proliferation of the mucosal epithelium in the basal layer of the tongue through the activation of the Wnt/β-catenin signaling pathway. The inhibitor of GSK3β SB216763 or SB415286 is studied only as a radioprotector primarily in animals because of its inclination to induce untoward effects in humans. The scientific community should further elucidate the side effects of the Wnt/β-catenin pathway to provide a potential therapeutic approach for preventing radiation-induced damage.

From the preceding discussion, we firmly believe that the Wnt/β-catenin signaling pathway activator should be developed as a potential therapeutic approach to prevent radiation-induced damage in the future. At present, confirming such protective effects is essential, particularly in the clinical setting (such as the approach, treatment window, and dosage of the inhibitor derived from the Wnt/β-catenin signaling pathway). However, considering the critical role of Wnt signaling in various cancer developments, the issue on specifically targeting the side effects of Wnt pathway-based drugs without increasing cancer risk should be addressed urgently.
